# Challenges in recruiting frequent users of ambulance services for a community paramedic home visit program

**DOI:** 10.1186/s12913-023-10075-9

**Published:** 2023-10-12

**Authors:** Mikayla Plishka, Ricardo Angeles, Melissa Pirrie, Francine Marzanek, Gina Agarwal

**Affiliations:** Department of Family Medicine, McMaster University, 100 Main St W, Hamilton, L8P 1H6 Canada

**Keywords:** Community paramedicine, Vulnerable populations, Frequent 9-1-1 callers, RCT, Recruitment, Paramedic

## Abstract

**Background:**

The Community Paramedicine at Home (CP@home) program is a health promotion program where community paramedics conduct risk assessments with frequent 9-1-1 callers in their homes, with a goal of reducing the frequency of 9-1-1 calls in this vulnerable population. The effectiveness of the CP@home program was investigated through a community-based RCT conducted in four regions in Ontario, Canada. The purpose of this current recruitment study is to examine the challenges met when recruiting for a community randomized control trial on high frequency 9-1-1 callers.

**Methods:**

Eligible participants were recruited from one of four regions participating in the CP@home program and were randomly assigned to an intervention group (*n* = 1142) or control group (*n* = 1142). Data were collected during the recruitment process from the administrative database of the four paramedic services. Whether they live alone, their parental ethnicity, age, reason for calling 9-1-1, reason for not participating, contact method, and whether they were successfully contacted were recorded. Statistical significance was calculated using the Chi-Squared Test and Fisher’s Exact Test to evaluate the effectiveness of the recruitment methods used to enroll eligible participants in the CP@home Program.

**Results:**

Of the people who were contacted, 48.0% answered their phone when called and 53.9% answered their door when a home visit was attempted. In Total, 110 (33.1%) of people where a contact attempt was successful participated in the CP@home program. Most participants were over the age of 65, even though people as young as 18 were contacted. Older adults who called 9-1-1 for a lift assist were more likely to participate, compared to any other individual reason recorded, and were most often recruited through a home visit.

**Conclusions:**

This recruitment analysis successfully describes the challenges experienced by researchers when recruiting frequent 9-1-1 callers, which are considered a hard-to-reach population. The differences in age, contact method, and reason for calling 9-1-1 amongst people contacted and participants should be considered when recruiting this population for future research.

## Introduction

The Community Paramedicine at Home (CP@home) program is a health promotion program, originally implemented in Ontario, Canada, that involves community paramedics visiting homes and conducting health risk assessments with frequent 9-1-1 callers and those who call for at least one ‘lift assist.’ Paramedics provide blood pressure, diabetes and fall risk assessments, health education/promotion and targeted referrals to in-house wellness programs and community resources. This program aims to reduce the frequency of 9-1-1 calls in vulnerable populations by providing health assessments and managing participants’ chronic conditions at home with their family doctor and with other community resources. These high frequency 9-1-1 callers could be defined as a hard-to-reach population with a lower quality of life and often have multiple health difficulties and disparities [1]. In addition, a recent study from Ontario demonstrates that frequent callers often have extreme mobility problems, pain, anxiety, and difficulties conducting usual activities, which can lead to falls [2]. Due to these mobility issues, older adults are at a high risk of falls and are at risk of having multiple fall-related 9-1-1 calls [3].

Additionally, poverty rates are higher in frequent 9-1-1 callers. Poverty produces multiple health care barriers due to unstable living arrangements, a failure to access required medications, and the inability to make healthy food choices. Consequently, manageable health conditions are left unmonitored and eventually require intervention through emergency services [2].

This paper explores participant recruitment of a community-based RCT conducted in four Ontario regions to evaluate the effectiveness of the CP@home program [4]. Community-based Randomized Controlled Trials (RCTs) are often used when evaluating health care interventions in a pragmatic way. Recruitment of participants into a community-based RCT can be very difficult and over 50% of these RCTs may fail to capture their intended populations [5]. The literature states that participants recruited for these RCTs often choose not to participate for a variety of reasons including time constraints, privacy concerns around medical information, failure to understand the purpose of the study and a lack of interest [6]. The challenges faced in recruitment for community RCTs are exacerbated in hard-to-reach populations, such as seniors, chronically-ill individuals, and individuals who frequently relocate [7].

This paper aims to describe the challenges met when recruiting for a community randomized control trial on high frequency 9-1-1 callers and to compare demographic characteristics, recruitment methods and reasons for calling 9-1-1 between those who could be contacted and those who could not be contacted by the RCT research team members, as well as between those who participated and those who did not participate in CP@home.

## Methods

This cross-sectional study utilized an observational design, based on recruitment data from a randomized controlled trial (RCT) conducted in four sites in Ontario beginning in May 2018 [4]. This current recruitment study examined participation rates based on demographics and method of contact, and the likelihood of a successful contact based on demographics and the primary reason for calling 9-1-1.

### Trial details

The CP@home program was a multi-site community-based randomized control trial (RCT) involving four paramedic services in Southern Ontario (Site 1, Site 2, Site 4) and Northern Ontario (Site 3) [4]. Community paramedics visited the homes of high risk 9-1-1 callers to conduct health assessments. Frequent 9-1-1 callers were defined as those who had called 9-1-1 at least three times in the previous 6 months. The main objective of the previously conducted RCT was to determine if registration in the CP@home program resulted in a change in the frequency of 9-1-1 calls and therefore ED visits compared to their own baseline and to the control group.

### Study population & eligibility criteria

The current study is a cross-sectional study of all individuals eligible to be recruited into the RCT. To be eligible for recruitment in the RCT, the study population was defined as individuals aged 18 and older residing in the community [8]. Participation in the RCT was voluntary.

#### Inclusion criteria

For the RCT, individuals were required to meet at least one of the following conditions:

1. had called 9-1-1 three or more times in the last 6 months and had called at least once within the previous month,
2. had called 9-1-1 for a lift assist within the previous month,
3. were directly referred by paramedics (identified through usual practice).

#### Exclusion criteria

Individuals living in long-term care facilities and individuals currently involved in a paramedic home-visit program or other paramedic-led frequent user intervention were excluded.

### Participant recruitment

Eligible participants were recruited from one of the four regions participating in the CP@home program. Each region's paramedic services identified eligible participants from the previous month and the information was sent to the Community Paramedicine Research Team. Individuals in long term care facilities who were involved in a similar paramedic led intervention were excluded.

High-frequency 9-1-1 callers meeting the study’s eligibility criteria were recruited into the community RCT and randomly assigned to an intervention group by the community paramedicine research team to receive scheduled health assessment visits from community paramedics or a control group who received usual care. The intervention group received the CP@home intervention and the control group received usual care. The CP@home intervention consists of a series of assessments to examine the participant’s overall health status, quality of life, and their social determinants of health. The program also connects participants with appropriate community resources. The aim of CP@home is to provide participants with resources, monitor their chronic conditions, and identify risk factors with the expectation that their health and quality of life will improve. CP@home includes many of the same aspects of CP@clinic [9], such as the overall health assessments, but also includes additional evidence-based screening assessments (e.g., neurologic, cardiac, psychiatric, and social isolation). Individuals in the intervention group were contacted by paramedics, either through phone call or a home visit, to determine their willingness to participate in the CP@home program. Their recruitment status was recorded.

### Data collection

Data were collected during the recruitment process from the administrative database of the paramedic services in the four study regions. The following data was recorded and used in the recruitment analysis: gender, age, parental ethnicity, whether the individual lived alone, their reason for calling 9-1-1, the outcome of the phone call, whether there was a home visit conducted to recruit the participant, whether consent was obtained, and reasons for not participating. A waiver of consent was obtained from the Hamilton Integrated Research Ethics Board (HiREB) during the intention-to-treat portion of the study, where consent was not required prior to randomization into the intervention group (attempt to offer the CP@home program) and control group (usual care) since all baseline data collected on participants was from de-identified secondary data sources. The consent process has been previously described in the protocol for CP@home [4].

For the purpose of this recruitment analysis, the availability of contact information was determined based on whether a contact attempt was made. Moreover, a phone call was the first line of contact to obtain initial verbal consent. Written consent was then obtained at the start of the first home visit. However, if verbal consent was not obtained because the individual was not able to be reached through a phone call, then a home visit was attempted to recruit participants, where written consent was obtained. For those who provided consent to receive the CP@home program, individual information was collected.

During the data coding process, the reason for not participating was grouped into nine categories: not interested, patient was deceased, unable to consent, LTC patient, issue had resolved, has sufficient support/care, unable to connect with patient, unknown, and other. Similarly, the reason for calling 9-1-1 was grouped into nine categories: pain/unwell, lift assist, trauma, behavioural problems/psychiatric, breathing difficulties, musculoskeletal, overdose, and a cardiac issue. Allocation to each category was determined by the authors based on the information entered into the database by the paramedic.

### Data analysis

To evaluate the effectiveness of the recruitment methods used to enroll eligible participants in the CP@home Program, descriptive statistics were conducted. Variables included demographic information (gender, age, living alone and parental ethnicity), recruitment methods (phone or door) and reasons for not participating. Raw counts, along with the corresponding prevalence, were reported for all demographics studied for everyone contacted and all participants. The chi-squared test was conducted to determine if there is a statically significant relationship between the method of contact and participants’ gender, age, and reason for calling 9-1-1. If there were less than 10 participants in at least one group, a Fisher’s Exact Test was used to calculate the statistical significance. Statistical significance was set at α = 0.05 and statistical analysis was conducted using SPSS 20.

## Results

**Fig. 1:**
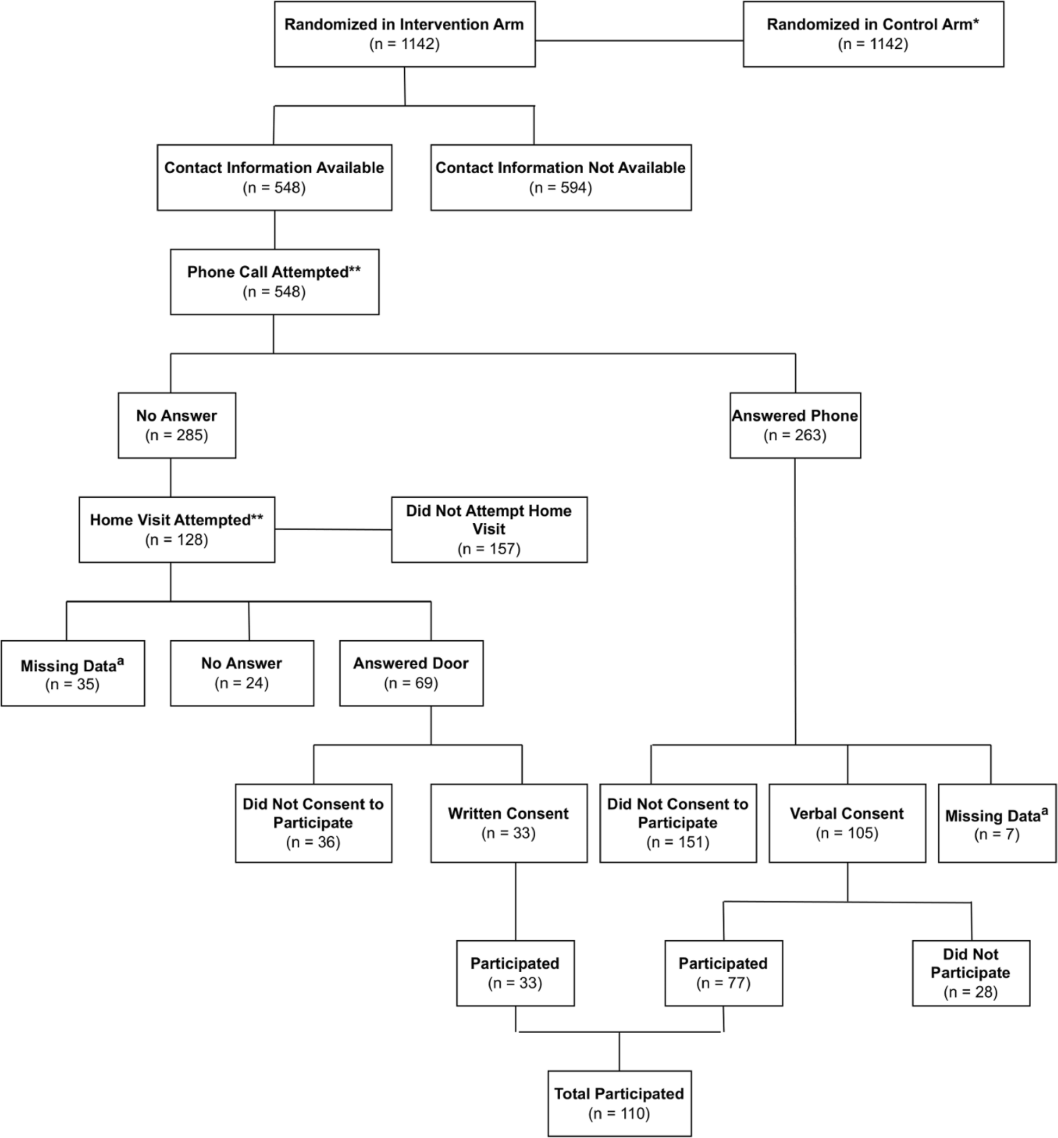
CP@home Recruitment Process * People randomized to the control arm are not part of the scope of this paper because there was no attempt to recruit for participation ** Some people were contacted both by phone and, if they were not able to be reached by phone, as a home visit ^a^ The outcome of the event was not recorded in the database

A flow diagram of the recruitment process for the CP@home program is shown in Fig. 1. There were 1,142 individuals randomized to the intervention arm, of which 548 (48.0%) of those individuals had contact information available. The availability of contact information was determined based on whether or not a phone call was attempted since this signified the availability of a phone number. Out of the phone call attempts, 263 (48.0%) answered their phone, which subsequently led to 105 (39.9%) of these individuals consenting to participate in the CP@home program. Consent for a home visit was obtained verbally, but written consent to participate in the study was obtained at the start of the first home visit. If there was no answer to the phone call, a home visit was attempted for 128 (44.9%) of individuals. Of the home visit attempts, 69 (53.9%) answered the door and, of these individuals, 33 (47.8%) provided written consent to participate in the CP@home program. There were a total of 110 participants in the CP@home program. Of those who participated, most were contacted through a phone call. Some (*n* = 28) individuals who verbally consented to participate did not actually proceed to schedule a home visit and participate in the program; there was no reason provided for 25 of these cases. For the 3 cases where a reason for non-participation was provided, one person was deceased when a home visit was attempted, one person lived in a group home, and one person was on the emergency list for long-term care (LTC).

When contact with an individual was made, 33 (47.8%) of the individuals who answered their door and 105 (39.9%) of individuals who answered their phone consented to participate in CP@home. The reasons that individuals did not participate are described in Table 1. The most common reason provided for non-participation for individuals who were visited in their home was that they have sufficient support / care and was reported by 13 (36.1%) people. The most common reason for non-participation for individuals who were contacted through a phone call was that they were not interested and was reported by 36 (23.2%) people. However, similar to individuals contacted through a home visit, having sufficient support / care was the second most common reason for non-participation in individuals contacted through a phone call, reported by 30 (19.9%) people.

**Table 1:** Reasons for non-participation in CP@home

Reason Provided	Home Visit (*n* = 36)	Phone Call (*n* = 151)
Not Interested	3 (8.33%)	33 (21.85%)
Deceased	0	14 (9.27%)
Unable to Consent	0	19 (12.58%)
LTC Patient	0	8 (5.30%)
Issue Resolved / Not Requiring Follow-Up	5 (13.89%)	12 (7.95%)
Has Sufficient Support / Care	13 (36.11%)	30 (19.87%)
Too Busy or Overwhelmed	4 (11.11%)	2 (1.32%)
Concerned About Burdening GP	1 (2.78%)	0
Feels They Do Not Need Any Services	4 (11.11%)	4 (2.65%)
Does Not Want to Be in a Study	1 (2.78%)	0
Too Nervous or Gets Stressed by New People or Situations	0	2 (1.32%)
Hung Up / No Time to Talk	0	4 (2.65%)
Moving	0	2 (1.32%)
Does Not Have Desired Service	0	1 (0.66%)
Did Not Believe Program Exists	0	1 (0.66%)
Not a Resident of Canada	0	1 (0.66%)
Does Not Want People Coming Into Their Home	0	1 (0.66%)
Unknown	5 (13.89%)	7 (4.64%)
Missing / Not Reported	0	10 (6.62%)

**Fig. 2:**
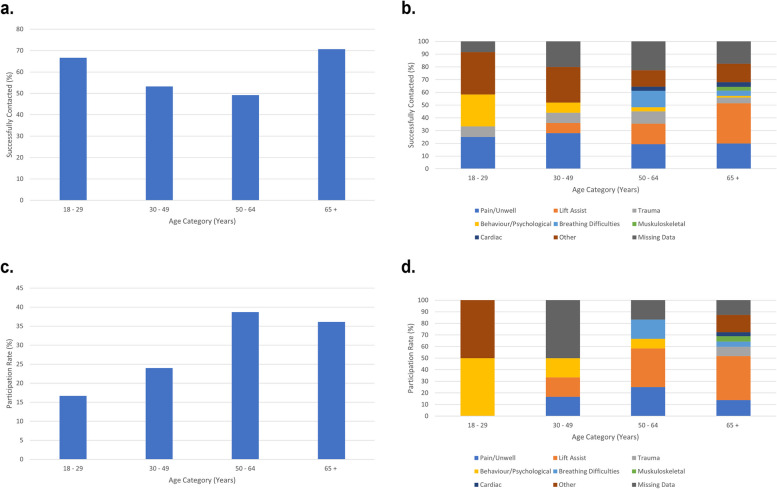
**a**. Proportion of Successful Contacts by Age Category. **b**. Distribution of Reasons for Calling 9-1-1 Amongst Successful Contacts by Age Category. **c**. Proportion of Participants by Age Category. **d**. Distribution of Reasons for Calling 9-1-1 Amongst Participants by Age Category

Of the 469 individuals with contact information available and age reported, 18 (3.8%) were 18–29 years old, 47 (10.0%) were 30–49 years old, 63 (13.4%) were 50–64 years old, and 341 (72.7%) were 65 or older. The age distribution and reason for calling 9-1-1 for individuals successfully contacted are presented in Fig. 2a and b, respectively. An individual was most likely to answer their phone or door if they were younger (18–29) or older (65 +). In both groups of contacts, over 65% of contact attempts were successful. This is greater than the middle age groups (30–49 and 50–64), where less than 55% of contact attempts were successful. For all contacted individuals, the most common single reason for calling 9-1-1 was behavioral/psychological in the youngest age group (18–29), being in pain or feeling unwell in the middle two age groups (30–49 and 50–64), and requiring a lift assist in the oldest age group (65 +). The least common reason for calling 9-1-1 across all age groups was a musculoskeletal reason.

Of the 107 participants with an age reported, 2 (1.9%) were 18–29 years old, 6 (5.61%) were 30–49 years old, 12 (11.2%) were 50–64 years old, and 87 (81.3%) were 65 or older. The age distribution and reason for calling 9-1-1 for CP@home participants is presented in Fig. 2c and d, respectively. Out of those successfully contacted, individuals aged 50–64 were most likely to participate in CP@home, where 40% consented to participate, followed by individuals aged 65 and older, where 36.1% of those who were successfully contacted consented to participate. Lift assist was the most common reason for calling 9-1-1 in participants in the oldest two age groups, followed by pain or being unwell. In the youngest age group of participants, the most common single reason for calling 9-1-1 was behavioral or psychiatric.

The distribution of reasons for calling 9-1-1 in the participants were different than the people successfully contacted. The proportion of lift assist patients in the 50–64 age group was much greater in the participants (13.3%) compared to lift assist patients who were successfully contacted in the same age group (6.5%). Moreover, trauma was provided as a reason for calling 9-1-1 across all age groups in individuals who were successfully contacted; however, trauma was a reason for calling 9-1-1 in only the 65 + age group of participants.

For CP@home participants, gender data was only available for Site 1 and was missing for Site 2, Site 3, Site 4. In Site 1, 24 (47.1%) participants were female, 25 (49.0%) participants were male, and 2 (3.9%) participants did not have their gender recorded.

In Site 2, Site 3 and Site 4 the majority of participants reported living alone. However, in Site 1 a slight majority reported living with a spouse based on their marital status. Data gathered about the ethnicity of the participant’s mother and father indicated that the vast majority of participants across all four study sites reported being white, with between 85 and 100% reporting a white parentage in Site 1, Site 2 and Site 3. Site 4 was the most diverse study site, with parentage being 70.6% white, 17.7% black, and 11.8% East Asian. These demographics are outlined further in Table 2.

**Table 2:** Demographics of CP@home participants

		**Site 1** **(Southern Ontario)** ***n* = 51** **n (%)**	**Site 2** **(Southern Ontario)** ***n* = 28** **n (%)**	**Site 3** **(Northern Ontario)** ***n* = 15** **n (%)**	**Site 4** **(Southern Ontario)** ***n* = 17** **n (%)**
Lives Alone	No	27 (52.94)	11 (39.29)	6 (40)	6 (35.29)
	Yes	24 (47.06)	17 (60.71)	9 (60)	9 (52.94)
	Missing	0	0	0	2 (11.76)
Ethnicity of Mother	White	44 (86.28)	28 (100)	14 (93.33)	12 (70.59)
	Black	1 (1.96)	0	0	3 (17.65)
	East Asian	1 (1.96)	0	0	2 (11.77)
	South Asian	4 (7.84)	0	0	0
	Other*	1 (1.96)	0	1 (6.67)	0
Ethnicity of Father	White	42 (82.35)	26 (92.86)	15 (100)	12 (70.59)
	Black	1 (1.96)	0	0	3 (17.65)
	East Asian	1 (1.96)	0	0	2 (11.77)
	South Asian	4 (7.84)	0	0	0
	Other*	2 (3.92)	2 (7.14)	0	0
	Missing	1 (1.96)	0	0	0

^*^Aboriginal, Latin American, West Asian

The differences in demographics and reasons for calling 9-1-1 between participants recruited through a phone call and participants recruited through a home visit are described in Table 3. When comparing participants contacted through a phone call and participants contacted through a home visit, there was a significant difference between participants who called 9-1-1 for a lift assist and those who called for another reason (*p* = 0.044). However, no other significant differences were observed between the two contact methods and gender, age, or the reason for calling 9-1-1.

**Table 3:** Differences between participants based on their contact status

		**Phone Call (*n* = 77)** **n (%)**	**Door Knocking (*n* = 33)** **n (%)**	***P* value**
**Gender**	**Female**	31 (40.26%)	2 (6.06%)	0.492^a^
	**Male**	32 (41.56%)	0	
	**Missing**	14 (18.18%)	31 (93.94%)	NA
**Age Ranges**	**18–29**	2 (2.60%)	0	1.00^a^
	**30–49**	5 (6.49%)	1 (3.03%)	0.666^a^
	**50–64**	8 (10.39%)	4 (12.12%)	0.750^a^
	**65 and older**	59 (76.62%)	28 (84.84%)	0.331^b^
**Reasons for calling 911**	**Lift Assist**	22 (28.57%)	16 (48.48%)	**0.044**^**b**^
	**Trauma**	4 (5.19%)	3 (9.09%)	0.426^a^
	**Behavior Problems**	3 (3.90%)	0	0.553^a^
	**Pain/unwell**	12 (15.58%)	5 (15.15%)	0.954^b^
	**Breathing Difficulties**	6 (7.79%)	1 (3.03%)	0.672^a^
	**Musculoskeletal**	3 (3.90%)	1 (3.03%)	1.000^a^
	**Cardiac**	3 (3.90%)	0	0.553^a^
	**Other**	10* (12.99%)	4** (12.12%)	1.000^a^
	**Missing**	14 (18.18%)	3 (9.09%)	NA

^*^Hypotension/Hypertension (1), Temporary Loss of Consciousness/Unconscious/Altered LOC/Post-ictal (2), Stroke/TIA (1), Diabetic Emergency/Low or High Blood Glucose (1), Infection/Infectious Disease (1), Non-traumatic Soft Tissue Problem (2), No Complaints (1), Leg Swelling (1)

^**^Hypotension/Hypertension (1), Seizure (1), Nausea/Vomiting/Diarrhea (1), Infection/Infectious Disease (1)

^a^Fisher’s Exact Test

^b^Chi-squared Test

## Discussion

This recruitment study has been able to successfully examine the factors that may contribute to a participant’s choice to be recruited into the CP@home program. It revealed many challenges and opportunities that future studies on frequent 9-1-1 callers could consider as part of their recruitment process.

Hard-to-reach populations, such as frequent 9-1-1 callers, often have low socioeconomic status; they often suffer from chronic health problems and/or mental illness, and may have precarious employment and living arrangements [2]. These characteristics of hard-to-reach populations make it challenging for these populations to participate and be represented in community-based healthcare research [1]. Given these factors, recruitment of frequent 9-1-1 callers may be difficult, as indicated by the recruitment rates in this study. Health programs that target this population should consider the fact that they can be hard to reach and take this into account during recruitment when deciding on the number of participants to contact.

We found that young adults (aged 18–29) and older adults (aged 65 and older) answered their phone or door more frequently than the middle age group. On the other hand, older adults aged 50–64 and 65 + who were contacted were most likely to participate in CP@home. These results should be considered in participant recruitment for future studies. For example, one should consider the need to oversample certain age groups that are harder to reach. Although the youngest age group was the most likely to respond to contact attempts, their participation rate was substantially lower than the older age groups suggesting that this group is highly contactable but has very little interest in participating. Their ability to be contacted could be a result of the frequent mobile phone usage in this group and not evidence of their interest in the program [10, 11].

Our recruitment analysis shows that older adults have a higher participation rate compared to younger age groups. Literature has shown that there are some common barriers in recruiting all ages of adults for health research and that language, culture, ill health and time and resources all play similar roles [12]. However there are some additional factors that older adults face that could influence recruitment rates. In our study, we hypothesize that the *intervention or program itself* was a huge draw for the older adult population. Having a health program at home could potentially be a benefit to this group as they are more likely to be in ill health [13, 14] and may be more likely to want to participate in a health program that does not require them to attend an appointment away from their house. For instance, older adults who have previously had the experience of a lift assist may be more likely to participate. Further, an at-home program may be more appealing for individuals who have a lack of resources to attend appointments. Older adults, who are more likely to have disabilities [15], may face barriers to attending appointments (e.g., transportation). In addition, social isolation and loneliness is a concerning issue in older adults living in social housing [2, 9]. Thus, this group may be lonely and welcome a visit from someone in their home. Lastly, older adults are more likely to show particular interest in the topics of health and medicine compared to younger adults [16].

Understanding the most effective way of contacting potential participants and the reasons for calling 9-1-1 is helpful in determining where to direct recruitment efforts for a home visit program. Among participants requiring a lift assist as a reason for calling, a significantly higher proportion were contacted through a home visit. Home visits were more likely to yield a response than phone calls as some people may be hesitant to answer the phone if they don’t know the number due to the high volume of scam calls [17]. This result may indicate that door knocking in order to request a home visit is an important method in recruiting lift assist patients who may have mobility challenges and may be more likely to be at home. Similarly, older adults who experience difficulties with mobility and are more likely to be lift-assist callers may find a home healthcare program particularly appealing since they do not need to endure the struggles of getting to and from medical appointments, but they are able to see a healthcare provider.

This paper had some limitations. The external generalizability of our study is limited because the vast majority of participants were of white ethnicity and therefore the findings may not be applicable to other ethnicities. Furthermore, there were discrepancies in data reporting between the four sites. Gender data was not reported for three of the sites, which limited the ability to examine recruitment differences by gender. Similarly, a reason for not participating was not recorded for most people who had consented to participate, but did not actually participate. This information could have been useful in providing an explanation into reasons for declining to participate after providing informed consent. The data was missing because paramedic services had difficulty in providing gender due to their restrictions with privacy and also the difficulties of extracting the information from their electronic medical records (EMRs). Lastly, reasons for calling 9-1-1 were categorized into groups, some of which were vague and non-descriptive (e.g., pain/unwell, other) which is how they are documented in the EMRs. This restricted the potential to compare more specific reasons for calling 9-1-1 across age groups of participants. However, each of these generic categories accounted for a small proportion of the total number of callers. Thus, the categorization method likely did not have any substantial impact on the analysis.

## Conclusion

This recruitment paper describes the difficulties often encountered when recruiting for a community randomized control trial on high frequency 9-1-1 callers. It can be difficult to reach this population due to a number of factors. Age, method of recruitment, and reason for calling 9-1-1 had impacts on the ability to contact and recruitment outcome, with older adults requiring a lift assist being more likely to participate in the home health program. These results can inform future studies of the challenges with recruiting frequent 9-1-1 callers by recognizing the implications of participants’ age, the method used to contact them, and their reason for calling 9-1-1. This study provides the foundation for further qualitative and quantitative exploration into the recruitment of older adults into a community randomized control trial.

## Data Availability

The data that support the findings of this study are not publicly available due to them containing information that could compromise participant privacy. De-identified, limited data will be shared by the corresponding author upon request.

## Abbreviations

RCT: Randomized Controlled Trial
ED: Emergency Department
CP@home: Community Paramedicine at Home
LTC: Long-Term Care
